# Clinical Review, and Hospital Reports

**Published:** 1842-10-01

**Authors:** 


### Clinical Review.

### UNIVERSITY HOSPITAL.

A Case of Aneurysms within the Skull, terminating in Apoplexy and Paralysis; with Clinical Remarks. By A. T. Thomson, M.D.*

John Morgan, æt. 49, of intemperate habits, was admitted into University Hospital on the 18th of January, 1842. It was impossible to obtain a correct history of his health, prior to this attack, but from the account obtained from his landlady, it appeared that some months prior to admission he had fallen in the street when tipsy, and cut the back of his head; since that time his manner was changed, he became garrulous, silly, and very irritable; a very small quantity of spirits would now make him tipsy.

On the morning of the day on which he was admitted, he felt unwell and remained in bed; in the afternoon he was found insensible, with his head hanging over the side of the bed. He had vomited a quantity of dark-coloured, somewhat stercoraceous fluid. He was sent to the hospital, about seven o'clock, p. m. in a comatose state. Pulse at the wrist scarcely perceptible; breathing laborious; left side of the body paralyzed, both as to sensation and motion. Mouth drawn to the right side. Right pupil more dilated than the left. Feet cold.

Soon after admission he was cupped and had a blister applied to the nape of the neck, the body was wrapped in blankets, an enema administered, and four grains of calomel with one of croton oil given as soon as possible after the enema. After the purging had ceased, he was ordered to take a four-grain calomel pill every fourth hour.

On the following morning sensation returned, but motion remained as before. Pulse 92, soft. Convulsive twitches of the mouth, and of the right arm and hand; and he could not protrude his tongue. To be cupped again on the temples, and to repeat the pills. He was also directed to take grs. v. of the sesquicarbonate of ammonia every 4th hour. Ice to the scalp.

This plan of treatment was pursued till the 25th, when he felt better. The head was cooler, the extremities of a more natural temperature. On the 27th, however, he became worse again, the pupils, especially the right, much dilated; breathing laborious; paralysis of motion continued, but the sensation was perfect on both sides.

On the morning of the 28th, the breathing was more laborious, the mucous râle louder. Extremities extremely cold; pulse at the wrist imperceptible. He died at a quarter of an hour past 12 o'clock, p. m.

*Examination, 26 hours after Death.*—The liver adhered anteriorly to the diaphragm; it was soft, easily torn, of a dark red colour, and presented at its anterior margin several yellowish granular masses. The lungs were partially adherent on both sides; the left heart slightly hypertrophied, but the valves were healthy. Kidneys small and granular. Stomach healthy.

*Brain.*—Vessels of the dura mater much gorged; pia mater vascular, especially on the left side; arachnoid opaque, with deposit of lymph on the left side. There was discoloration of the anterior lobe of the right hemisphere; and a patch of extravasated blood, about two inches in diameter, under the right parietal

* London and Edinburgh Monthly Journal, July 1842.

bone, and beneath the pia mater; there was also a smaller patch of a similar description on the left hemisphere. About two ounces of bloody fluid at the base of the brain. Middle and anterior lobes on both sides adhering by false membranes at the base. The arachnoid was opaque in that situation, and an aneurysm, a little larger than a hazel-nut, was found on the trunk of the carotid, where it gives off the arteria cerebri media, and another small one on the curve of the artery. The right portion of the circle of Willis was wanting. On the same artery of the opposite side, there was also an aneurysm, nearly globular; the branches of the vessel going to the anterior lobe of the brain were enlarged, and their coats thickened. The anterior lobe on the same side as the large aneurysm was also softened almost to a pulp. The basilar artery was dilated, besides being white, opaque, and its coat much thickened. The anterior lobe on the opposite side was softer than usual.

*Clinical Remarks.*—The diagnosis as to the cerebral origin of the paralysis could not be mistaken, although the condition of the brain, which had induced the attack, could not possibly have been conjectured. The immediate attack was evidently apoplectic; the imperfect history renders the exciting cause of the seizure doubtful, but it is probable that the fall which the patient had six months before, must have predisposed to apoplexy. It is not improbable that softening had then begun, and was slowly proceeding. There was, however, no doubt that the commencement of both the apoplexy and the paralysis was simultaneous; that extravasation had taken place was rendered probable from the form of paralysis being hemiplegia. In this case there was serous effusion at the base of the brain, but none into the ventricles—owing evidently to the state of the cerebral vessels, and the termination of inflammation of the meningeal coverings of the brain.

The coagulable lymph thrown out upon the left hemisphere was much greater than that upon the right; this is to be accounted for by the obstruction of the supply of arterial blood to that hemisphere, owing to the large aneurysm: on which also, probably, the softening depended.

In reviewing the case, there can be little doubt that both the apoplexy and the paralysis were the result of inflammation set up in the brain, previously predisposed to take on such a state. This predisposition depended on the derangement of the circulation caused by the fall, and the consequent formation of the aneurysms, a state of things most likely to occur in an intemperate person. It is probable also that the immediate cause of the inflammation and extravasation was drunkenness.

The softening, which existed chiefly in the anterior lobe of the right hemisphere, was the result of the defective supply of blood to that part caused by the large aneurysm. The substance of the left hemisphere was softer than natural, arising also from the state of the vessels. The senses and intellectual faculties were impaired, as usually occurs in the first period of softening, from obstruction of the vessels of the brain. In the second period, the symptoms of inflammatory softening and that from defective circulation or obliteration, are the same. We must regard, in this case, the serous effusions as altogether secondary, the result of the meningeal inflammation.

The case is interesting in a pathological point of view. It adds another to the recorded cases of aneurysm within the cranium; and shows that these aneurysms may attain a large size. It proves moreover that life may be sustained under very considerable derangement of the brain, and that organic changes within the skull are less likely to terminate quickly, than inflammatory action set up in the organ.

With respect to the treatment in the present case, and in every apoplectic attack connected with arterial excitement, the first object is to lessen the impulse of the blood on the cerebral arteries; and if the pulse does not contra-indicate the free abstraction of blood, it should be carried to its full extent. It is necessary also to subdue the torpid state of the bowels; and here Dr. Thomson thinks that the employment of *elaterium* has been too much neglected in the treatment of simple apoplexy. No other purgative, he thinks, is so likely to maintain the beneficial effect procured from a full bleeding, without that serious depression of power, which a repetition of copious venesection always produces. The calomel was pushed with the view of aiding the blood-letting, and the purging in reducing the inflammatory action. It is, however, unnecessary to comment on the treatment; the prognosis was unfavourable from the commencement, and much experience was not requisite to pronounce that the case would terminate fatally.

---

### PENNSYLVANIA HOSPITAL.

### A Case of Inguinal Aneurysm treated successfully by a Ligature to the External Iliac Artery. By Edward Pearce, M.D.*

John Erwin, aged 28, was admitted into the Pennsylvania Hospital, July 17th, 1841, with inguinal aneurysm on the right side. Four months previously, after a fall received in wrestling, he had severe pain in right groin, which however left him in the course of a few days. Two months afterwards the pain in the groin returned, and he then observed, for the first time, a small tumor of the size of a walnut, which has continued to enlarge, and is accompanied with so much pain as to incapacitate him from walking.

Upon examining the patient in the hospital, a pulsating tumor was found in the situation of the right femoral artery, extending from one inch above Poupart's ligament to three and a half inches below it. The transverse diameter of the tumor was four inches. The skin over the tumor was reddened: deep-seated and severe pain. There was also much pain at the inside of the knee; the whole limb was swollen; sensibility natural except upon the anterior surface of the thigh.

In six days time all signs of local inflammation had disappeared, and the skin had resumed its natural appearance; the tumor had increased rapidly in size, but the pulsation in it was not so strong. It was then determined to tie the external iliac.

July 24th. A curved incision, four inches in length, with its convexity directed towards Poupart's ligament, was made through the skin, commencing an inch and a half above the anterior superior spine of the ilium, and terminating half an inch above the situation of the external ring. The tendon of the external oblique was then divided upon a director, bringing into view the lower edge of the internal oblique and transversalis muscles, which were separated from Poupart's ligament with the handle of the scalpel. The peritoneum was easily raised up, and the artery was felt beating distinctly, but faintly, contrasting strongly with the violent vibration of the tumor. The artery, which appeared healthy, was separated from the vein by the finger nail, and a silk ligature applied, with great facility, as high up as possible, so as to allow sufficient space for the formation of a coagulum above the epigastric artery. The pulsation of the tumor was immediately arrested. The lips of the wound were brought together by two strips of adhesive plaister, and dressed with lint spread with cerate. The operation lasted eighteen minutes.

Six o'clock, P.M. Foot cold and moist, the rest of the limb natural, but somewhat painful. Pulse 74. Ordered Sol. Morph. ʒij.; foot enveloped in carded wool.

On the following day, there was violent pain in the groin, which was relieved by fomentations, and the administration of morphia. Temperature of the right thigh ninety-six, of the left, ninety-three.

---

* Medical Examiner, Philadelphia, April 9. 1842.

July 27th. Both limbs of the same temperature; sensibility natural everywhere, except the last phalanges of right toes, which remain cold. A small red spot at the inner side of the patella is very painful, prominent and soft; the tumor is also painful and inflamed.

July 28th. Much irritability of the bladder; tumor smaller, less painful. Ordered flax-seed tea, and a large cataplasm over pubic region.

July 29th. Passed a comfortable night, has made water but twice since yesterday; wound suppurating freely; two-thirds of the incision have united by the first intention. Simple dressing to the wound.

The patient continued to improve without any unfavourable symptoms; the tumor becoming smaller and firmer till August the 24th, thirty days after the operation, when the ligature came away, having a large loop.

September 30th. Wound completely cicatrized; tumor half of its former size. The patient walks about the room, and can bear his whole weight upon the affected side.

November 24th. Discharged from the hospital; is able to return to his work. The tumor is now about the size of a walnut.

*Remarks.*—Owing to the rapid progress of the aneurysm, it was not deemed proper to apply pressure above the tumor with a view of dilating the collateral branches, as recommended by several eminent surgeons; and it may well be questioned whether the benefit resulting from dilatation of the vessels which are to nourish the limb, will ever compensate for the greater danger incurred by an increase of the tumor and inflammation of the surrounding tissues. The same reasons may be urged against the recommendation to promote a cure by pressure over the tumor, as exemplified in a case of inguinal aneurysm, treated by Dr. Post, where the patient refused the application of a ligature.

His surgeon then applied a compress to keep up a constant pressure upon the tumor. Under this treatment the aneurysm diminished for a time, but then increased rapidly, while severe pain and considerable local inflammation and tumefaction of the upper part of the thigh supervened. These symptoms were finally relieved by a removal of their cause, and a resort to cold applications and evacuants. When the patient at last submitted to an operation, it was found impracticable to separate the peritoneum, usually so easy, owing to the adhesions that had taken place between that membrane and Poupart's ligament from the previous inflammation. The surgeon was obliged to cut through the peritoneum in order to apply the ligature: consequently the patient was exposed to the additional hazard of inflammation of that membrane. Fortunately the termination of the case was favourable.

In this case, with the exception of the irritation of the bladder on the fifth day no unpleasant symptoms of any kind occurred during the whole course of treatment, which was unnecessarily prolonged by the ligature not having been tied sufficiently tight. This was manifested by the large size of the loop, and it satisfactorily accounts for the retention of the ligature for thirty days.

---

### LEEDS GENERAL INFIRMARY.

### REPORT OF CASES TREATED BY MR. T. P. TEALE.*

CASE I.—*Strangulated Hernia—Operation—Sac not opened.* John Midgeley, æt. 69, admitted 14th of March, 1842, with a large strangulated hernia, occupying the inguinal canal and scrotum on the left side. The portion

* Provinc. Medical Journal, July 2.

of the tumor within the canal feels hard and cylindrical, and is separated from the scrotal portion by a strongly-marked depression in the site of the external ring, which evidently exerts a firm constriction on the tumor; the abdomen is distended and tympanitic.

States that he has had a hernia there for seven years; has never worn a truss, but has always been able to return it himself until the 12th March, when it came down and he was unable to replace it. On the 13th, he had one small fæcal evacuation, and occasionally vomited.

The taxis, with the warm bath, having proved unsuccessful, a large clyster was ordered to be given immediately; a bladder containing the " freezing mixture " to be applied over the tumor and two calomel and colocynth pills to be given every two hours.

In the evening, though the symptoms were not urgent, as the bowel remained strangulated, it was determined to operate.

Having divided the skin, superficial fascia, and fascia of the cord, a director was passed under the external ring, which embraced the tumor very tightly, and a few fibres at its upper boundary were divided; after which, by a little manipulation, the protruded parts were replaced within the abdomen, a gurgling sound accompanying their return. The edges were brought together by two or three sutures, over which a compress of lint was applied.

15th. The bowels acted freely two hours after the operation, and the patient felt perfectly relieved. On the following day, on removing the dressings, the wound was found to be united throughout its whole extent.

17th. Erysipelatous blush at the upper extremity of the wound, which has re-opened, and looks sloughy; no tenderness of the abdomen. A strong solution of caustic to be applied to the inflamed skin, and poultice to the skin.

18th. Erysipelas has not extended. Wound discharges freely.

April 15th. Wound healed. A truss, with a large but slightly convex pad, has been applied. Discharged.

Case II.—*Compound Fracture of the Cranium.—Depression of Bone.—Laceration of Longitudinal Sinus.*

Mary Rushforth, æt. 16, admitted on the 2nd of April, with severe injury of the head; having fallen, in an epileptic fit, from a room in which she was working, through an opening in the floor, about 12 feet, into the room below, her head pitching upon the iron rim of a large wheel.

On examination, it was found that a wound of the scalp, three inches in length, extended over the right parietal bone, parallel to and a little behind the coronal suture, terminating a little to the left of the median line. The right parietal bone was fractured, and depressed in the same situation as the wound in the scalp; the depressed bone was broken into several fragments, and its anterior edge pressed deeply upon the dura mater. She vomited occasionally, but was perfectly sensible.

Mr. Teale having applied the trephine in three places, raised or removed the depressed portions of bone; on raising that portion situated nearest the mesial line, it was found that the longitudinal sinus had been wounded, and on removing the bone, a profuse torrent of venous blood flowed over the patient's head. Though the hæmorrhage was speedily stopped by a compress of lint, enough blood was lost to render the patient blanched and faint. The wound was dressed with lint smeared with cerate.

On the following day slight re-action took place, but no symptom of cerebral disturbance occurred. On the 6th, she had an epileptic fit of short duration, after which she was restless, with heat and dryness of skin and quickness of pulse. Wound to be dressed with creosote lotion; pil. cal. cum col. and salines.

8th. Yesterday, at three P.M. she had another fit, which was followed by copious venous hæmorrhage from the wound. A compress of lint was again introduced and retained by a bandage.

10th. Wound looking healthy, except that the dura mater appears to be in a sloughy state, and that there is a tendency to protrusion of the brain. A graduated compress of lint was applied.

17th. Slight hæmorrhage has occurred several times, but has now ceased; she has not had any epileptic attack for several days. Wound looking healthy.

May 14th. Wound rapidly granulating. Has had a slight epileptic attack this morning. To take a fourth of a grain of nitrate of silver four times a day.

June 1st. Wound healed. Discharged.

CASE III.—*Strangulated Ventral Hernia.—Operation.*

Richard Morton, æt. 69, of plethoric habit, was admitted into the Infirmary on the 1st of June, 1842, labouring under symptoms of obstruction of the bowels.

He states, that after violent exertion about two years ago, a tumor formed at the left side of the abdomen, which has never since then entirely disappeared.

At five o'clock this morning, he began to suffer from severe pains in the abdomen, with frequent vomiting. He is now much exhausted, vomiting large quantities of black fluid, like coffee-grounds; much perspiration; pulse 118, intermittent; abdomen tender, more especially in the left iliac region, where a flattened oval tumor, about three inches in length is perceptible; this tumor is situated beneath the aponeurosis of the external oblique, which forms a tense and smooth covering of the tumor. The bowels have not acted since yesterday. A dose of cal. and col. immediately, and ol. ricini in an hour afterwards. A large clyster to be injected.

The injection brought away a small quantity of fæcal matters, but as the vomiting and pain in the abdomen continued, Mr. Teale determined to operate.

An incision was made in the long axis of the tumor, exposing the aponeurosis of the external oblique, which was next divided, when the hernial sac, covered by a rather thick layer of tissue, presented itself. On opening the sac, a coil of large intestine, highly vascular, and a small portion of omentum were exposed. The constriction was found to consist in an opening in the internal oblique and transversalis muscles, presenting a sharp tendinous edge at its mesial border, where it was contiguous to the linea semilunaris.

The opening was dilated at its upper border, the intestine was returned into the abdomen, but the omentum, having contracted extensive adhesions, was allowed to remain in the sac.

In half an hour after the operation the patient had a copious fæcal evacuation, and the abdomen became rather softer. Pulse 120, very feeble. On the following day he died.

*Sectio cadaveris.*—A small portion of the sigmoid flexure of the colon had again descended into the sac, where it had formed adhesions, these, however, were so lax as to allow of the gut being readily pushed up. The mucous membrane of the whole of the large intestine, from the cœcum to the protruded portion of the sigmoid flexure, was quite black and of soft consistency. The interior of the cœcum and colon contained black fluid similar to that which had been vomited; the portion of sigmoid flexure lying in the sac was considerably injected, but its mucous membrane did not exhibit very much discoloration; below this part the gut appeared perfectly natural. The small intestines were natural, nor was there any effusion in the peritoneal cavity, nor deposition of lymph upon the serous surface.

### NEW YORK HOSPITAL.

### Scrofulous Ulcer of the Neck, penetrating the Inferior Thyroid Artery—Death from Hæmorrhage.*

John Redmond, æt. 28, was admitted into the New York Hospital, April 30, 1841, with scrofulous enlargement of the glands of the neck. This disease, which was of long standing, involved the whole chain of glands on both sides, and in several places had gone on to suppuration followed by indolent ulceration. His general health was much impaired.

Under the use of iodine in various forms, the system became much improved, though the external disease was not benefited, indeed during the summer it made considerable progress. Fresh abscesses formed in various parts, and those which had been long opened, degenerated into foul burrowing ulcers, undermining the skin, and in some places burrowing deep among the muscles. He was in this state when suddenly a slight hæmorrhage took place from one of the largest and deepest of the sinuses, situated on the left side of the neck, over the middle portion of the sterno-cleino-mastoid muscle. The bleeding was slight and stopped spontaneously. During the next night, bleeding again took place to a much greater extent, and of a bright arterial colour. A graduated compress of lint, and moderate pressure completely checked the hæmorrhage.

On the following morning, on removing the dressings, a large stream of arterial blood spouted out, rendering it obvious that some large vessel had been opened by ulceration. The sinus was immediately stuffed full of lint, and firm pressure applied by means of the fingers. A considerable quantity of blood was lost.

A consultation of the surgeons was then called. On removing the compress in order to examine the wound, a gush of blood immediately took place, spouting out full six feet from the bed, and in a very considerable stream. The compress was instantly replaced, but not before so much blood had been lost as to reduce the pulse to a mere thread. As it was impossible, in consequence of the violence of the bleeding, to secure the vessel in the wound, and as there was too much swelling of the neck to determine accurately the exact source from which the hæmorrhage proceeded, it was determined to tie the common carotid, in the hopes that it might be either that vessel or one of its branches that was affected.

On performing the operation, in the usual situation of the sheath of the vessels, a large mass of fibrine was found, adhering to all the tissues in that region, and confounding them so as to render it extremely difficult to distinguish one from the other. After careful dissection, what appeared to be the sheath of the vessel was exposed and divided. A cylindrical body of the size and colour of the artery was then brought into view, and a ligature passed under it. Though no distinct pulsation could be felt, the operator, as well as the other surgeons, being convinced that it was the artery, the ligature was tied, without however, arresting the flow of blood. From this it was evident, that the subclavian or one of its branches was the injured vessel, but the patient was in too low a state to admit of any further operation.

Compresses were applied so as to control the hæmorrhage, and stimulants administered. During the night, however, the compress unfortunately became displaced, and so much blood was lost that in two hours the patient expired.

A careful examination was made after death. The ligature, which was thought to have been placed round the carotid artery, was found to enclose only a band of organized lymph situated upon the sheath of the vessels which were perfectly

* New York Medical Gazette, Feb. 9, 1842.

healthy. On removing the coagulum and cleansing the ulcer, it was found to have extended backwards and downwards towards the subclavian artery, the upper surface of which, just within the thyroid axis, it had reached within a quarter of an inch. At the bottom of the wound the thyroid axis lay exposed, and from it given off the inferior thyroid, running upwards and forwards, and destroyed by ulceration in one half of its circumference for the space of an inch. The ulceration had involved slightly the thyroid axis itself. The inferior thyroid, beyond the ulcerated opening, was completely obliterated, showing previous inflammation of the artery. Indeed it would appear probable that the whole of the thyroid artery had been obliterated before ulceration commenced, and would soon have been destroyed without danger, had not the ulceration reached the thyroid axis, and this, not being protected by coagula, gave rise to the fatal hæmorrrhage. The other arteries of the body were healthy.

---

### HOTEL-DIEU.

### Clinical Practice of M. Roux.*

We shall select from this report some remarks with regard to amputations and the excision of joints.

### I. Amputations.

During the two years 1840, 1841, M. Roux performed 35 capital amputations, as follows.

*Amputations of the Thigh.*

| | | | | | | | |
|---|---|---|---|---|---|---|---|
| In 1840 | .. 8 | .. | Cured | 4 | .. | Died | 4 |
| In 1841 | .. 8 | .. | Cured | 2 | .. | Died | 6 |
| Total.. | .. 16 | | | 6 | | | 10 |

In one of these amputations of both thighs was performed, at an interval of a month.

*Amputations of the Leg.*

| | | | | | | | |
|---|---|---|---|---|---|---|---|
| In 1840 | .. 6 | .. | Cured | 4 | .. | Died | 2 |
| In 1841 | .. 6 | .. | Cured | 3 | .. | Died | 3 |
| Total.. | .. 12 | | | 7 | | | 5 |

*Amputations of the Arm.*

| | | | | | |
|---|---|---|---|---|---|
| In 1840 | .. .. .. | Cured | 1 | .. | Died 2 |
| In 1841 | .. .. .. | | 0 | | 0 |

*Amputation of the Fore-Arm.*

| | | | | | |
|---|---|---|---|---|---|
| In 1840 | .. .. .. | Cured | 1 | .. | Died 0 |
| In 1841 | .. .. .. | Cured | 3 | .. | Died 0 |

These two years afford therefore 35 capital operations; the result, 16 deaths; 18 cures.

This result is stated to be favourable in comparison with previous years. Since 1836, 178 amputations had been performed at the Hôtel Dieu; of these 104 died and 74 were cured.

---

* Gazette Médicale de Paris.

Out of these 178 amputations, 63 were of the thigh, of which 43 proved fatal, and 20 were cured.

With regard to the nature of the cause rendering the removal of the limb necessary, it appears that in 11, the lesions were acute, as gun-shot wounds, gangrene, &c.

| | | | | | | | |
|---|---|---|---|---|---|---|---|
| Of the Thigh.. | .. | 4 | .. | Died 4 | .. | Cured | 0 |
| „ Leg .. | .. | 3 | .. | „ 3 | .. | „ | 0 |
| „ Arm .. | .. | 2 | .. | „ 2 | .. | „ | 0 |
| „ Fore-arm .. | | 2 | .. | „ 0 | .. | „ | 2 |
| | | 11 | | 9 | | | 2 |

In 24, the lesion was chronic, as white swelling, organic affection of the bones, &c.

| | | | | | | | |
|---|---|---|---|---|---|---|---|
| Of the Thigh.. | .. | 12 | .. | Died 5 | .. | Cured | 6 |
| „ Leg .. | .. | 9 | .. | „ 2 | .. | „ | 7 |
| „ Arm .. | .. | 1 | .. | „ 0 | .. | „ | 1 |
| „ Fore-arm .. | | 2 | .. | „ 0 | .. | „ | 2 |
| | | 24 | | 7 | | | 16 |

*Remarks.*—The ratio of mortality, as exhibited by these tables, does certainly appear to us to be extraordinarily high, much higher than it is in England. In a report of the results of amputations, at University College Hospital, published a short time ago in the Medico-Chirurgical Transactions, it appeared that, during a period of about six years and a half, 66 amputations had been performed there, of these 56 were cured and only 10 died; and out of 364 amputations, collected by Dr. Machardy,* only 83 deaths took place.

### II. Excision of Joints.

No surgeon in France appears to have so exerted himself to bring this operation into favour, as M. Roux. The difficulties and length of the operation itself, however, and the time during which it is necessary to wait for a definite result, for a complete cure, have proved serious obstacles to its being much employed. M. Roux thinks that he owes a considerable portion of the success which he has met with in performing this operation, to an important modification adopted by him, since August 1840, namely in making a T incision by the side of the limb, instead of the H incision usually employed by other surgeons. This modification renders it more easy to dress the wound without disturbing the limb, or causing it the slightest motion. The elbow is the joint to which this operation is most applicable. The following is the report of 6 cases of excision of this joint, performed by M. Roux.

No. 1.—Caries of all the bones of the left elbow, in a woman aged 41. Complete success. Re-establishment of all the motions.

No. 2.—White-swelling of the right wrist, with abscess, numerous fistulous sinuses. Young man, aged 22. Excision of all the bones entering into the composition of the joint. Very remarkable success.

No. 3.—Excision of left elbow, in a man aged 59, for white swelling, accompanied by abscess, sinuses, enormous fungous growths; success.

No. 4.—Excision of the right humero-cubital articulation, for caries of the three bones. Death 16 days after the operation, from extensive erysipelas.

* Vide Med. Chirurg. Review, No. 71, Page 172.

No. 5.—White swelling of the left elbow; excision; complete success in a man aged 26.

No. 6.—Excision of the right elbow in a man, æt. 40, for white swelling. The patient is still in the Hôtel Dieu, the wound being not yet quite healed.

---

### CASE OF LESION OF SENSIBILITY AND MOTION IN A PERSON LABOURING UNDER SYPHILIS. By PAUL WILLIAM SWAIN, Esq.*

Robert Roseovar, aged twenty-eight, contracted a venereal disease when at the age of 20, for which he was treated by an empiric. He soon after had an attack of rheumatism, which lasted, with varying intensity, for some months.

Three years ago a severe pain fixed itself in the region of the occiput and upper cervical vertebræ, and soon afterwards these parts began to enlarge. It appears that he took very little notice of these symptoms for some time, until their increasing intensity compelled him to seek medical assistance.

Accordingly he was directed to take iodine, and was treated for venereal periostitis; this had the effect of diminishing the enlargement in the occipital region, but considerable tumefaction remained, and this still continues.

Eighteen months ago, the pain in this tumefaction became much aggravated and extended down the back. The patient now, for the first time, began to suffer from slight twitchings, principally in the lower extremities, but occasionally in other parts of the muscular system. These gradually increased until they became violent spasmodic actions of extension and flexion. The lower extremities seemed to elongate, and were then suddenly flexed upon the abdomen. Occasionally the arms were forcibly thrown above the head, though the upper extremities have always been more under the control of volition than the lower. The limbs were slightly benumbed, especially the right leg, but the sense of touch on the surface seems to have been throughout unimpaired; he experienced a sense of impending suffocation; the breathing was often hurried: the least emotion increased his spasms; he had frequent priapism and spontaneous emission.

Nothing seems to have been done for the poor fellow at this stage of his suffering. His friends, thinking that he was bewitched, consulted a professor of the black art.

The lower extremities lost the intensity of their spasmodic action, but by degrees were drawn up in a state of tonic spasm, so as to be firmly flexed upon the body. About this time he was recommended to employ daily considerable extension of the contracted extremities, and on one occasion of the performance of this operation, early in June last, the thigh-bone, probably affected by venereal taint, gave way at its middle third, in which part the patient had for some time felt considerable pain.

It was at this period that Mr. Swain first saw the case, and, being struck with its peculiarities, determined to examine it with attention.

Every means have been devised to keep the fractured thigh in a state of extension, but as the patient could only lie on his back, or partially on one side, and excoriations took place at the points where the extending forces had their bearing, nothing like a sightly cure of the broken bone could be effected. The large adductor muscles have drawn the lower end of the fractured bone almost to a right angle with the upper portion, whose point very nearly penetrates the integuments and skin of the thigh, which is nearly five inches shorter than its fellow, nor is there at the present moment any union between the broken portions of bone.

---

* Prov. Med. Journ. August 27.

The patient experiences no pain of any consequence, nor has there been any severe suffering from the first moment of the fracture. The following is a short summary of his condition :—

Slight emaciation about the legs: appetite good: bowels open daily: pulse natural: mind unaffected and in full power: the head is slightly thrown back, but he can move it laterally to a considerable extent.

When the bed-clothes are removed so that the cold air comes in contact with the body, the lower extremities are still further drawn up, until the heels rest upon the nates; after a short time they gradually relax, and assume their usual state, but tickling the soles of the feet reproduces the spasmodic contraction. On the occurrence of any intense or sudden emotion, the arms partake of the same kind of flexion, although in general he can help himself to all he requires with his hands.

He is unable to retain his urine any time, but he never wets his bed during sleep, nor has he at present any priapism.

The patient being still alive, we can only speculate on the exact pathology of this interesting case, but we are considerably assisted by the details of a very similar one mentioned by Dr. Marshall Hall, in his late volume on "Diseases and Derangements of the Nervous System," when a post-mortem examination discovered a nodule of bone in the dorsal region of the spinal canal. In this case we may fairly infer, in like manner, that a venereal exostosis is attached to the inner surface of one of the upper cervical vertebræ, pressing upon the spinal-marrow, and giving rise to the abovementioned symptoms.

---

### Surgical Cases and Observations. By James Syme, Esq.*

*Nasal Polypus.*—Andrew Lawson, æt. 47, was admitted into the Hospital on the 23rd of April. In the early part of the present year, he observed a growth in the left nostril, which gradually increased, obstructing the passage and bleeding from time to time. It at length protruded externally, bleeding profusely on several occasions. The tumor distended the nostril, had a brownish red colour, with soft friable consistence, and bled under the slightest touch.

No doubt existed as to the nature of the disease, which was obviously malignant, and negatived any attempt at removal by evulsion. The simple mucous polypus may easily be removed by the forceps, but the vascular pulpy growths which originate from the osseous texture do not admit of complete eradication, except by taking away the bone from which they spring; and as the part affected is usually inaccessible, from involving the sphenoidal or ethmoidal cells, it is hardly ever possible to effect this. The only prudent course, therefore, in such cases is, to abstain from interference, as even clearing the nostrils has, in some instances, proved fatal, by exciting inflammation of the parts within the cranium.

The patient accordingly, after having had the projecting portion of the tumor cut off, was informed that nothing more could be done for him, and left the hospital as incurable. He soon afterwards returned to say that the bleeding had increased, and became very desirous of having an operation performed. On re-examination the previous opinion as to the nature of the disease was confirmed, but some favourable circumstances were detected. Though the nostril was dilated, the root of the nose and nasal bones did not show the slightest appearance of enlargement; there was no pain or uneasy feeling in the region of the frontal sinuses; the eyes were natural as to position and vision; the morbid growth was confined to one nostril; and it seemed to be connected with the walls of the

* London and Edin. Monthly Journal, September.

cavity near the external orifice. As there thus appeared reason to conclude that the disease originated either from the inferior spongy bone, or some other accessible part, Mr. Syme determined to try what could be done for the poor man's relief.

An incision was made through the upper lip, from the nostril downwards to the mouth, and the flaps separated so as to examine the attachment of the tumor. It then appeared that the growth proceeded from the septum, by a narrow neck not larger than a four-penny piece, immediately above the connection of the cartilage to the bone, and that there was consequently no difficulty in rooting out the disease. The septum was then cut through a little way above the lower margin, so as not to interfere with the columna, the bone divided, and the remaining cartilaginous attachments separated. The surfaces of the wound were then brought together, and retained by sutures. In the course of a few days there was hardly any perceptible trace of the operation, and the patient has since continued perfectly well.

*Remarks.*—This case shows how careful we should be before condemning a patient as incurable; it also affords an example of malignant disease in the nasal cavity, originating from a very unusual source; and it proves the advantage of gaining additional freedom of access to the nostril, by dividing the lip, instead of cutting through the *columna*, or slitting open the *ala*, neither of which modes would have afforded so much space, or probably have left so little deformity. Some years ago Mr. Syme laid open the nostril in the last-mentioned way in order to extract a large fibrous polypus, but did not succeed; afterwards he removed the superior maxillary bone, as the only means of relieving the patient from the disease. The morbid growth for which this severe operation was undertaken, is fortunately of very rare occurrence. It is distinguished by firmness or tenacity of texture, resembling that of the ligamentous structures, strong and extensive adhesions to the surface of the bones composing the nasal cavity, and tendency to bleed, causing frequent and profuse epistaxis. In 1840, M. Flaubert of Rouen, removed the superior maxilla, for the detachment of a fibrous polypus, and the same operation has recently been performed by Professor Mott, of New York, with success.

In all these cases the bone was perfectly sound, and was removed merely to afford room foı separating the tumors: these, therefore, are very different from the ordinary affection of a malignant nature, in which the growth springs from the interstices of the bones, and, except in such very peculiar circumstances as occurred in this case, does not admit of any beneficial operation.

---

### STATISTICS OF OPERATIONS.

### REMARKS ON AMPUTATION. By ALEXANDER KING, Licentiate of the Royal College of Surgeons in Edinburgh.

Mr. King, the author of the Remarks upon Amputation, was induced to direct his attention to the subject from observing the difference of results between the operations in Glasgow and the Country, and particularly from witnessing the mortality in the Glasgow Infirmary.

Mr. King first touches on the subject of immediate and secondary amputations after injuries, and agrees with those (the majority) who advocate the former. Yet statements are as contradictory as can well be imagined. The following Tables bear upon the subject.

1. Mr. King introduces a table of Mr. Alcock's, it is as follows :—

| | PRIMARY. | | | SECONDARY. | | |
|---|---|---|---|---|---|---|
| | No. | Died. | Mortality. | No. | Died. | Mortality. |
| Mr. Guthrie's Series at Thoulouse:— | | | | | | |
| Upper Extremity ................ | 7 | 1 | 7. | 16 | 4 | 1 in 4 |
| Lower Ditto .................... | 41 | 9 | 4.5 | 37 | 18 | 2. |
| Mr. Alcock's Series in Spain and Portugal:— | | | | | | |
| Upper Extremity ................ | 12 | 1 | 12. | 12 | 2 | 6. |
| Lower Ditto .................... | 21 | 4 | 5.2 | 21 | 10 | 2.1 |
| Messrs. Hayward and Norris's Amputation for Injuries received into Civil Hospitals:— | | | | | | |
| Upper Extremity ................ | 17 | 3 | 5.6 | 11 | 1 | 11. |
| Lower Ditto .................... | 26 | 8 | 3.2 | 25 | 11 | 2.2 |
| Messrs. Hayward and Norris's Amputations for Chronic Disease:— | | | | | | |
| Upper Extremity ................ | 10 | | | | | |
| Lower Ditto .................... | 48 | 8 | 6. | | | |
| | 58 | 8 | 7.2 | | | |

Now, from a Table of amputations performed in the Edinburgh Royal Infirmary from 1st July, 1839 to 1st July, 1841, it appears that there were 69 amputations, with 50 recoveries and 19 deaths. Of 50 male patients 15 died—of 19 female patients 4 died, leaving the balance of recoveries in favour of the females. The primary amputations were 10 with 9 deaths! The secondary were 33 with 9 deaths. This is in favour of secondary amputations.

The following Table from the Massachusetts Hospital leans to the same side.

*Amputations performed at the Massachussetts General Hospital,—reported by Dr. Hayward, (since opening of Institution.)*

| | PRIMARY. | | | SECONDARY OR OTHERWISE. | | |
|---|---|---|---|---|---|---|
| | No. of Cases | Cured. | Died. | No. of Cases | Cured. | Died. |
| Thigh.. ........... | 6 | 3 | 3 | 28 | 23 | 5 |
| Leg ................ | 5 | 3 | 2 | 19 | 15 | 4 |
| Shoulder .......... | | | | | | |
| Arm ............... | — | — | — | 4 | 4 | — |
| Fore-arm .......... | 2 | 2 | — | 4 | 3 | 1 |
| | 13 | 8 | 5 | 55 | 45 | 10 |

Grand Total, 68 Cases, 53 recoveries, 15 deaths.

It seems hard to decide the matter from Tables.

To proceed with Mr. King's "Remarks." He reprobates a very long knife for the first incisions—an ordinary-sized Lisfranc's knife is best, as there is no necessity for changing it in order to cut upon the bone.

Mr. King, we are glad to see, is no party to the *humbug* of affecting to despise the tourniquet upon every occasion. No doubt it can be dispensed with when there is good assistance, but, if properly managed, it is itself a very excellent assistant. Mr. King observes :—

"When enlarged lymphatic glands lie over the course of the blood-vessels, the Tourniquet is decidedly preferable to compression with the hand. I have seen an injurious loss of blood occasioned by trusting to compression when this state of parts existed."

Mr. King makes some sensible observations on the *Circular and Flap Operations* :—

"The method of operating by flap, which appears to have originated with Mr. Lowdham of Oxford, and is now generally adopted in this part of Scotland, is, after time has been afforded for a fair trial of its merits, very generally admitted to be more easily and rapidly executed than the circular, and consequently less painful to the patient; and usually the reunion is more speedy and certain, the stump is better covered, less conical, and in every respect better suited for all after purposes. It is alleged that the blood-vessels are secured with great difficulty after the flap operation, in consequence of being cut obliquely; but this appears to be a theoretical objection. Until Mr. Liston's removal to London, the flap operation was almost exclusively confined to Scotland, and the circular is still generally preferred in England, on the Continent, and the United States of America. I have always adopted the flap operation, because I think the ease and rapidity with which it can be performed, recommend it strongly to young Surgeons; but from what I have seen in the practice of friends, and in hospitals, I do not think that the double flap, the oval, or any of their combinations deserve the exclusive preference which Mr. Liston appears to give them. Malgaigne, a very excellent authority, in his *Operative Surgery*, prefers on the whole the circular method; and Dr. Lawrie, to whose practical lectures on Surgery I owe much, is also favourable to the circular. Dr. Ballingall, in his *Military Surgery*, says :—

'I know of no comparative estimate of the results of amputation performed by the circular incision, and by the double flap, which will enable to decide their respective merits by the test of experience.'—*Page* 368.

In the truth of this observation I fully concur. The tables given by Mr. Alcock, in his lectures in the *Lancet* (1840-41), page 647, are very unsatisfactory, and the numbers are too small to settle the various questions involved."

No candid person of good sense and experience can join, we think, the exclusive partisans of either method. The circular operation, if well performed, is undoubtedly a successful one, and, no doubt, the flap operation is successful too. One man will prefer one and excel in it—another man will choose the other. Mr. King thinks the circular operation best calculated for the middle of the arm and upper part of the leg.

Mr. King touches on the question of amputating the leg just below the tuberosity of the tibia or lower. He observes that the lower the amputation, the less the risk. The chief reason for preferring the high amputation, is the interference of a long stump with the operations of the common wooden leg. But in Glasgow *that* is not employed, another sort of apparatus being had recourse to. It consists of a hollow wooden box, to which a short wooden pin is attached, and is made to press on a pad fixed round the leg below the knee. When such an apparatus is used, the patient has the free and unrestrained use of the knee-joint, and walks with as great freedom and ease as if he had only an anchylosed ankle joint; whereas with the pin, he requires to drag the leg behind him, on account of the action of the muscles being interfered with, by the bent state of the joint. This apparatus is equally well calculated for all other purposes as well as walking. It is worth while trying this in our London hospitals.

In amputation of the thigh, Mr. King prefers anterior and posterior to lateral flaps. For he says :—

"When the flaps are laterally formed the bone is apt to be raised by the action of the muscles, or in the act of dressing the stump, and left uncovered at the upper angle of the wound; and the pressure of the lower edge upon the pillow, separates the incised surfaces, prevents adhesion by first intention, and leaves a large suppurating cavity. When the line of incision is transverse, the surfaces are pressed together, and maintained in apposition around the bone by the weight of the parts themselves, and a most favourable opportunity is thus afforded for immediate union. By keeping one angle somewhat lower than the other, the pus is allowed as free an exit as when the flaps are formed laterally."

Mr. King reprobates, in just terms, the absurd way of dressing a stump, yet, perhaps, too prevalent. Straps, plaisters, compresses, bandages, are piled one upon the other, with the effect of heating, inflaming, promoting the formation of matter, and locking it up when formed. All this is barbarous surgery. Mr. King's method is the following :—

"I have been in the habit of bringing the incised surfaces together, and keeping them in that state by two or three stitches, assisted by a narrow strap of plaster between each. Care is taken that sufficient openings are left for the escape of pus, &c. between each stitch and plaster. Over the line of incision is applied a piece of lint spread with soft ointment. An equable pressure is kept up over the whole limb by the application of a bandage, commencing from above, and carried downwards by circular folds to nearly the edges of the incision, but avoiding the front of the stump, in case of retarding the escape of matter. The limb is then placed on a pillow, on a level with the body, with one edge of the wound slightly depressed, and covered in such a manner as may best suit the season of the year."

We are not convinced of the propriety of any circular bandage at all. If it makes any compression it must interfere, pro tanto, with the return of the venous blood. If it makes no compression, we do not see what object it can serve.

Mr. King alludes with dissatisfaction to the plan of allowing the surface of the stump to glaze, as it is called, before bringing the edges together. This was originally introduced by Dupuytren. Mr. King observes :—

"It is alleged that this method diminishes the risk of secondary hæmorrhage, and is consequently safer for the patient than that usually followed. That hæmorrhage does occasionally occur, when the patient, from being in a state of collapse on the table, has become heated in bed, is unquestionably true; but if the most important blood-vessels have been carefully secured, its occurrence is too unfrequent to justify a surgeon in resorting to such a harsh expedient, as I consider dressing *a deux temps* to be. Notwithstanding what has been said to the contrary, I am satisfied from the few cases in which I have seen it tried, that the patient suffers nearly as much from the continued anxiety and alarm, joined with the pain of the dressing at the late period, when the parts have become swelled, —more or less inflamed, and acutely sensitive, as from the operation itself. When secondary hæmorrhage does unfortunately occur, the patient will suffer less from the separation of the edges, and the application of a ligature to the bleeding vessel, than from the alternative which Mr. Liston has suggested. Every body knows that very slight pressure is often sufficient to arrest hæmorrhage from arteries of the third and fourth importance, and the mere pressure of the flaps will close up many vessels from which more or less blood will issue if left covered with wet cloths. Weak, debilitated patients frequently fall into a tranquil and refreshing sleep, after the fatigues of an operation; an event so highly desirable cannot happen when the dressing is delayed, as the patient's mind is in a state of perpetual excitement till he is satisfied all is over; he cannot be expected to know all the steps of the operation, and after having borne the pain of an amputation, he must be excused for exhibiting more or less incredulity, when told that the subsequent dressing is attended with no pain. The plan has been fairly tried by many practical men, and has been very generally condemned."

We confess that we are of Mr. King's way of thinking. If the vessels are properly secured at first, there is little danger of hæmorrhage. We suspect the dressing *à deux temps* finds favour because it is rather conducive to *éclat*. The patient is got quickly off the table. But this is a pitiful motive.

Mr. King speaks with amazement, and we are not much less astonished ourselves, at the attempt lately made by Mr. Phillips and others to detract from the advantages of union by the first intention. To abandon this would indeed be to retrograde in surgery. Mr. King argues the question very ably, and, to our minds, very satisfactorily. The only shadow of a reason for preferring secondary union, is when a copious discharge has existed before the operation is performed. Mr. King's remarks on this head are very forcible and just.

"It is alleged, that the most pernicious results are likely to follow the immediate union of the stump, when extensive suppuration has existed in the limb before its removal. This doctrine appears very theoretical; its existence in practice is denied by surgeons of the highest standing, and the cases adduced in its support by Mr. Alcock are of the most unsatisfactory description. Mr. Guthrie, no doubt, gives it as the result of his experience, that fatal secondary deposits of matter in chest, liver, &c. &c. are more common after secondary amputation, and when suppuration has been profuse previous to the operation, than after primary, and appears to attribute this to the sudden arrest of the discharge; but he grants 'the continuance of the discharge would soon have destroyed the patient.' It must be borne in mind, that an operation is never performed at this late period, except the symptoms have become more aggravated, or some new cause of alarm presented itself, demanding the removal of the limb. In many of these cases the morbid action has commenced before the knife is resorted to, and would have terminated fatally, although no operation had been performed. For instance, a compound fracture is put up in the ordinary manner, and every thing goes on favourably for a time, when without any assignable cause, the patient has a rigor followed by profuse perspiration,—the appearance of the limb becomes less favourable, and the discharge more profuse,—a consultation consider amputation advisable,—the patient, a few days after the operation, dies, and dissection displays large secondary deposits; but had no operation been performed, the termination of the case and the morbid appearances would have been, in all likelihood, the same. Operations performed after well-established rigors are almost constantly fatal, secondary inflammation having previously existed. I do not recollect of having seen one case of compound fracture or amputation recover after the supervention of severe rigors. The question of immediate or secondary union cannot be settled by statistical data, except the physical and dynamic causes which were in operation be thoroughly understood. Out of ninety-two cases, treated by Percy by immediate union, eighty-six were cured in twenty-six days; of seventy-five treated in the same method by Lucas, five died; of six operated on by Pelletan, and treated by union by granulation, one recovered."

The remainder of the Pamphlet is chiefly occupied with the remedies for due ventilation in the wards of a hospital, and an exposé of the bad state of the Glasgow Infirmary. But these subjects require no further notice.

